# Decoration of intramyocellular lipid droplets with PLIN5 modulates fasting-induced insulin resistance and lipotoxicity in humans

**DOI:** 10.1007/s00125-016-3865-z

**Published:** 2016-02-10

**Authors:** Anne Gemmink, Madeleen Bosma, Helma J. H. Kuijpers, Joris Hoeks, Gert Schaart, Marc A. M. J. van Zandvoort, Patrick Schrauwen, Matthijs K. C. Hesselink

**Affiliations:** Department of Human Movement Sciences, NUTRIM School for Nutrition and Translational Research in Metabolism, Maastricht University Medical Centre+, 6200MD Maastricht, the Netherlands; Department of Human Biology, NUTRIM School for Nutrition and Translational Research in Metabolism, Maastricht University Medical Centre+, Maastricht, the Netherlands; Department of Cell and Molecular Biology, Karolinska Institutet, Stockholm, Sweden; Department of Genetics and Cell Biology—Molecular Cell Biology, Cardiovascular Research Institute Maastricht, Maastricht University Medical Centre+, Maastricht, the Netherlands; Institute for Molecular Cardiovascular Research IMCAR, Universitätsklinikum Aachen, Aachen, Germany

**Keywords:** Fasting, IMCL, Lipid droplet size, Lipotoxicity, Perilipin 5

## Abstract

**Aims/hypothesis:**

In contrast to insulin-resistant individuals, insulin-sensitive athletes possess high intramyocellular lipid content (IMCL), good mitochondrial function and high perilipin 5 (PLIN5) levels, suggesting a role for PLIN5 in benign IMCL storage. We hypothesised a role for PLIN5 in modulating fasting-mediated insulin resistance.

**Methods:**

Twelve men were fasted for 60 h, before and after which muscle biopsies were taken and stained for lipid droplets (LDs), PLIN5 and laminin. Confocal microscopy images were analysed for LD size, number, PLIN5 association and subcellular distribution.

**Results:**

Fasting elevated IMCL content 2.8-fold and reduced insulin sensitivity (by 55%). Individuals with the most prominent increase in IMCL showed the least reduction in insulin sensitivity (*r* = 0.657; *p* = 0.028) and mitochondrial function (*r* = 0.896; *p* = 0.006). During fasting, *PLIN5* gene expression or PLIN5 protein content in muscle homogenates was unaffected, microscopy analyses revealed that the fraction of PLIN5 associated with LDs (PLIN5+) increased significantly (+26%) upon fasting, suggesting PLIN5 redistribution. The significant increase in LD number (+23%) and size (+23%) upon fasting was entirely accounted for by PLIN5+ LDs, not by LDs devoid of PLIN5. Also the association between IMCL storage capacity and insulin resistance and mitochondrial dysfunction was only apparent for PLIN5+ LDs.

**Conclusions/interpretation:**

Fasting results in subcellular redistribution of PLIN5 and promotes the capacity to store excess fat in larger and more numerous PLIN5-decorated LDs. This associates with blunting of fasting-induced insulin resistance and mitochondrial dysfunction, suggesting a role for PLIN5 in the modulation of fasting-mediated lipotoxicity.

***Trial registration*:**

trialregister.nl NTR 2042

**Electronic supplementary material:**

The online version of this article (doi:10.1007/s00125-016-3865-z) contains peer-reviewed but unedited supplementary material, which is available to authorised users.

## Introduction

Excess lipid can be stored ectopically as triacylglycerol in lipid droplets (LDs) (e.g. in skeletal muscle). This is often associated with compromised myocellular insulin sensitivity [1–3]. Rodent studies revealed that a high-fat diet increases intramyocellular lipid (IMCL) content and promotes insulin resistance [4]. Acute elevation of lipid availability in humans profoundly augments IMCL storage and insulin resistance [5]. How excess fat storage in muscle affects insulin sensitivity, however, is not yet clear.

Excess fat in muscle is stored in LDs containing predominantly neutral lipids like triacylglycerol and cholesteryl esters. LD size [6], subcellular distribution [7–9] and composition [10] and interaction of LDs with other cellular organelles like mitochondria [11] have all been postulated to contribute to the observed association between IMCL storage and insulin resistance. Paradoxically, stimulating muscle fat storage by overexpressing diacylglycerol-*O*-acyltransferase-1 (DGAT1) profoundly augments IMCL storage [12] without compromising insulin sensitivity [13, 14]. Moreover, endurance-trained athletes have high levels of IMCL while being very sensitive to insulin [15] and are less sensitive to acute lipid-induced insulin resistance upon lipid infusion [5]. In searching for a unifying factor in conditions of maintained insulin sensitivity despite the presence of high levels of IMCL, we noticed that in all conditions when insulin sensitivity was maintained even though IMCL was high or elevated, the gene expression or protein content of perilipin 5 (PLIN5) was increased [13, 16, 17].

PLIN5 is a member of the perilipin LD coat proteins and is predominantly expressed in metabolically active tissues [18–20] where it appears to regulate oxidative LD lipolysis [21] and hence affects LD size. To examine the effect of PLIN5 on lipid-induced insulin resistance we overexpressed PLIN5 in rat skeletal muscle and observed a profound increase in intramyocellular LD content while insulin-stimulated glucose uptake was not impaired [22]. In line with this observation, it has recently been shown that whole-body deletion of PLIN5 in mice reduced insulin-mediated glucose uptake [23]. Of the other perilipin family members present in muscle, perilipin 2 (PLIN2) has also been reported to promote the retention of lipids in LDs and to prevent high-fat-diet-induced reductions in insulin sensitivity [24] whereas perilipin 3 (PLIN3) has recently been associated with fat oxidation in cultured muscle cells [25]. To date, PLIN5 is the only member of the perilipin family that has been directly linked to LD turnover and fat oxidation in multiple models of insulin resistance [26]. Thus, we focused on PLIN5 and hypothesised that elevation of PLIN5 contributes to the maintenance of insulin sensitivity under conditions of an abundance of lipids. Under pathophysiological conditions like type 2 diabetes, insulin resistance of adipose tissue results in disinhibition of adipose tissue lipolysis by insulin. Thus, the pathological state of type 2 diabetes creates a situation in which glucose, insulin and plasma NEFA are elevated. Although the putative effects of insulin on lipolysis of myocellular LDs has not been studied in detail, it has been reported that insulin decreases gene expression of the major adipose triglyceride lipase (ATGL) [27]. Moreover, transcriptional activation and phosphorylation of members of the perilipin family are reportedly affected by elevated insulin and glucose levels [28]. We hence studied the putative role of PLIN5 in the physiological regulation of insulin sensitivity, in a model in which lipid overload was physiologically induced and in which blunted insulin sensitivity could not be attributed to secondary effects of long-term pathophysiological consequences of insulin resistance, like hyperglycaemia or hyperinsulinaemia. Therefore, we employed our studies in a model of prolonged fasting (60 h) in humans, which we previously published [29]. In this study, prolonged fasting resulted in a profound decrease in insulin sensitivity and an increase in IMCL content, while insulin and glucose levels were in the low end of the physiological range. Interestingly, rodent data indicate that—at least in cardiac muscle—a prolonged fast was paralleled by increased myocardial fat content and induction of PLIN5 [19]. Therefore, we hypothesised a role for PLIN5 in modulating fasting-induced insulin resistance.

As we previously observed that genetically promoting PLIN5 resulted in increased LD size [22], we combined studies in whole-muscle homogenates with detailed quantitative confocal fluorescence microscopy-based morphometric data permitting analysis of individual LDs under fed and fasted conditions.

## Methods

### Ethics statement

The study protocol was approved by the institutional Medical Ethical Committee. All participants (*n* = 11) gave written informed consent prior to participating in the study. This study has been registered in the Nederlands Trial Register (www.trialregister.nl) with registration number NTR 2042. The plasma data presented in this paper originate from previously published work [29].

### Participants and study protocol

A detailed protocol of the present study has previously been published elsewhere [29]. In brief, 12 young, normoglycaemic lean men were studied in the fasted (60 h fast) or fed state, in a randomised crossover design with a 2 week washout period in between. None of the participants was engaged in sports > 2 h per week. To ensure compliance to the intervention, the entire study was performed in a respiration chamber. In the fasted state only energy-free drinks were consumed. In the fed state participants were fed in energy balance (with 50%, 35% and 15% of energy intake consumed as carbohydrates, fat and protein, respectively).

Post-intervention (60 h fast or fed), a hyperinsulinaemic–euglycaemic clamp was performed (40 mU m^−2^ min^−1^ insulin combined with infusion of [6,6-^2^H_2_]glucose to measure rates of glucose disposal (rate of disappearance [Rd]). Prior to the clamp, a biopsy was taken from the vastus lateralis muscle. One portion was processed for mitochondrial capacity assays (high-resolution respirometry to assess ADP-driven state 3 respiration in permeabilised muscle fibres using octanoyl-CoA, glutamate and succinate as substrates). Maximal uncoupled respiration was measured upon carbonyl cyanide-4-(trifluoromethoxy)phenylhydrazone titration (FCCP). Oxygen flux was normalised to mitochondrial content based upon mtDNA copy number) [29]. Another portion of the sample was frozen directly in melting isopentane, and stored at −80°C for qRT-PCR analysis, western blotting and quantitative (immuno)fluorescence microscopy. Muscle insulin sensitivity was expressed as insulin sensitivity index (S_I_-index) and was calculated as (Rd insulin stimulated − Rd basal) / (plasma insulin × plasma glucose). Confocal immunofluorescence is based upon nine individuals. Characteristics of all participants are presented in Table 1.

**Table 1:** Characteristics of participants (*n* = 12)

Variable	Mean ± SEM
Age (years)	23.6 ± 1.0
Body weight (kg)	78.5 ± 2.5
Fat-free mass (kg)	65.9 ± 1.8
Height (m)	1.86 ± 0.02
BMI (kg/m^2^)	22.6 ± 0.5
Maximal aerobic capacity ([ml O_2_] kg_FFM_^−1^ min^−1^)	57.5 ± 1.5

FFM, fat-free mass

### Quantitative real-time PCR

RNA was isolated from approximately 30 mg muscle tissue essentially according to Chomczynski et al [30]. Quantitative real-time RT-PCR was performed as described previously [31, 32]. Primers and probes are presented in electronic supplementary material (ESM) Table 1. Gene expression data for *PLIN5* were normalised over *RPLP0*.

### Western blots

Western blots were performed using antibodies against PLIN5 (Progen GP31; Progen Biotechnik, Heidelberg, Germany) and SR-actin (A-2172; Sigma, St Louis, MO, USA). Infrared-tagged secondary antibodies (IRDye; LI-COR, Lincoln, NE, USA) were used to visualise and quantify the relevant protein bands (Odyssey Infrared Imaging system; LI-COR Biosciences, Westburg, Leusden, the Netherlands).

### Histochemical analysis

Fresh cryosections (7 μm) of samples from the fed and fasted state were cut and thaw-mounted on a single glass slide to minimise variety in staining intensity. Initial quantification of IMCL was performed by Oil-Red-O staining [33]. To allow valid LD morphometry, sections displaying freezing damage were discarded. As a result, full morphometric analyses were performed on 3,595 ± 522 and 4,766 ± 489 LDs per participant (*n* = 9) in the fed and fasted state, respectively. For quantification of PLIN5, sections were fixed in 3.7% vol./vol. formaldehyde in PBS for 30 min, washed for 5 min with PBS, blocked for 45 min with blocking buffer (150 mmol/l NaCl, 20 mmol/l Tris pH 6.8 and 2% wt/vol. BSA) and permeabilised with 0.25% wt/vol. Triton-X 100 (648466; Merck, Darmstadt, Germany). Subsequently, sections were washed with PBS for 5 min followed by a 1 h incubation with primary antibodies against laminin (L9393; Sigma) and PLIN5 (GP31; Progen Biotechnik, Heidelberg, Germany) in blocking buffer at room temperature. LDs were visualised using Bodipy 493/503 (Molecular Probes, Leiden, the Netherlands). Sections were incubated for 1.5 h along with the appropriate secondary antibodies conjugated with Alexa Fluor 405 or Alexa Fluor 647 (Invitrogen, Groningen, the Netherlands) at 37°C and mounted with Mowiol (5886; Merck).

### Confocal image acquisition and analysis

Multiple sliced Z-stacks were acquired on a Leica TCS SPE confocal microscope using a ×63 1.3 N.A. oil immersion objective with a 1.1 optical zoom at 2,048 by 2,048 pixels resulting in a pixel size of 77 by 77 nm. Laminin–Alexa Fluor 405, Bodipy 493/503 and PLIN5–Alexa Fluor 647 were imaged using the 405, 488 and 635 nm laser lines, respectively. Emission was detected at 415–460, 500–560 and 650–750 nm for, respectively, Laminin–Alexa Fluor 405, Bodipy 493/503 and PLIN5–Alexa Fluor 647. To permit valid quantification of the fluorescence signal from PLIN5–Alexa Fluor 647, pixel saturation was prevented. Z-stacks were acquired for deconvolution purposes (Huygens Essential software; Scientific Volume Imaging, Hilversum, the Netherlands) and analysed using ImageJ (NIH, Bethesda, Maryland, USA) [34]. Intensity of PLIN5 staining was analysed on the non-deconvoluted images. LDs coated with PLIN5 (co-localisation with PLIN5) and devoid of PLIN5 were quantified using a custom-written routine in Matlab R2012a (The Mathworks, Natick, MA, USA).

### Statistical analyses

Results are presented as mean ± SEM. Statistical analyses were performed using SPSS version 21.0 (SPSS, Chicago, IL, USA). Statistical comparisons between conditions were performed using paired *t* tests. Pearson’s correlation coefficients were used to describe the linear association between variables. *p* < 0.05 was considered statistically significant.

## Results

### Plasma variables

We previously reported a significant increase in circulatory NEFA levels (∼ ninefold) and a significant decrease in plasma glucose values, insulin levels and insulin sensitivity (glucose infusion rate and S_I_-index) upon fasting [29]. These data indicate that fasting created the warranted condition of profound insulin resistance and increased circulatory NEFA in the absence of hyperglycaemia or hyperinsulinaemia for the present study.

### IMCL content and insulin resistance

**Fig. 1:**
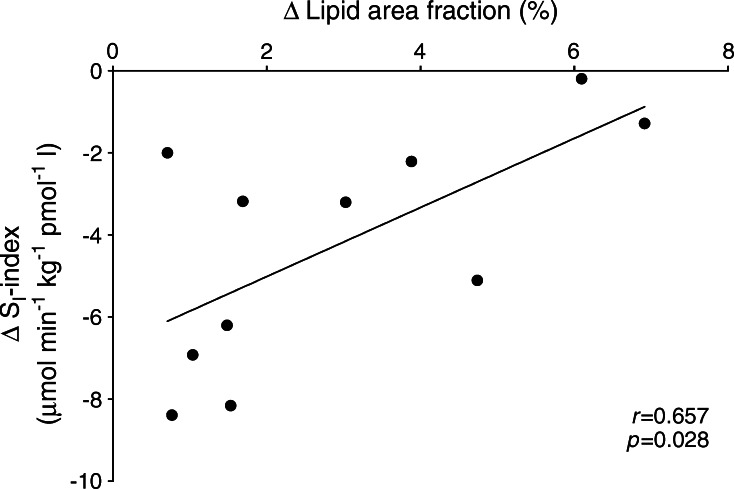
Correlation of the change in IMCL content and S_I_-index (μmol min^−1^ kg^−1^ pmol^−1^ l) upon prolonged fasting (*n* = 11), suggesting that promoting the capacity to store lipids as IMCL ameliorates fasting-induced insulin resistance. This correlation appears to be driven by PLIN5-coated LDs (Fig. 6a, b)

Fasting significantly augmented IMCL content (1.67 ± 0.32 arbitrary units [AU] in the fed state; 4.60 ± 0.72 AU after fasting) [29]. Interestingly, the increase in IMCL (IMCL in the fasted state minus IMCL in the fed state) upon fasting correlated positively (*r* = 0.657; *p* = 0.028) with the decrease in the S_I_-index (S_I_-index in the fasted state minus the S_I_-index in the fed state) (Fig. 1), suggesting that promoting IMCL storage capacity ameliorates fasting-induced insulin resistance.

### Morphometric analysis

**Fig. 2:**
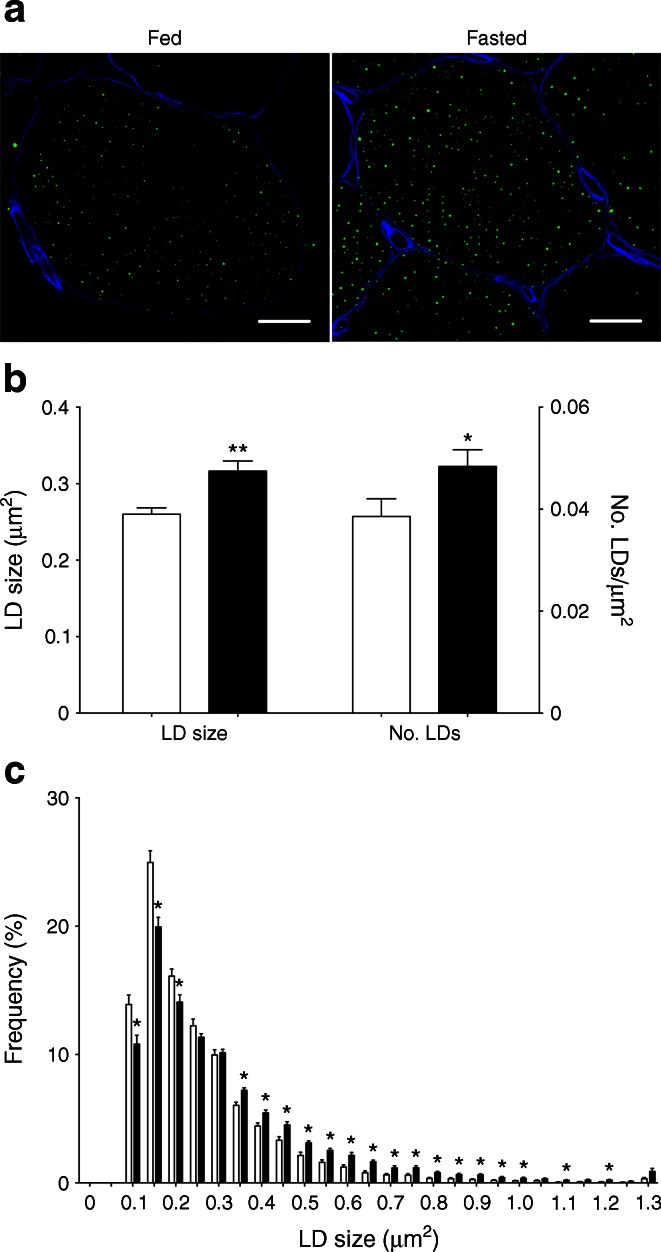
LD size and number increased upon prolonged fasting. (**a**) Representative images of muscle fibres from the fed and fasted state (magnification × 70; scale bar, 15 μm). LDs are stained in green and cell membranes in blue. (**b**) Quantification of LD size and number relative to cell area. (**c**) Frequency distribution of LD size. Data are based upon examination of 3,595 ± 522 and 4,766 ± 489 LDs per participant (*n* = 9) in the fed and fasted state and presented as mean ± SEM. *p < 0.05 and **p < 0.01 vs fed state. White bars, fed state; black bars, fasted state

As changes in LD size [6] may contribute to the development of insulin resistance we employed microscopy to examine LD size and number. Fasting promoted LD size (0.26 ± 0.01 vs 0.32 ± 0.01 μm^2^; *p* < 0.01) and number (0.039 ± 0.004 vs 0.048 ± 0.003 μm^−2^; *p* < 0.05) (Fig. 2a, b). LD size distribution analyses revealed a drop in the percentage of small LDs (≤0.20 μm^2^) upon fasting, whereas the percentage of large LDs (≥0.35 μm^2^) significantly increased (Fig. 2c).

### PLIN5 content on individual LDs

**Fig. 3:**
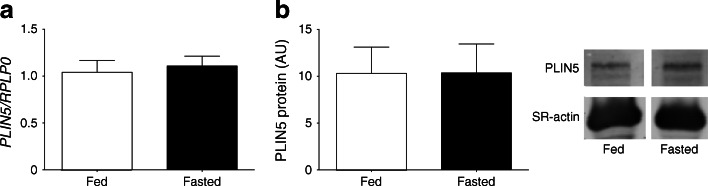
*PLIN5* gene expression (*n* = 12) (**a**) and PLIN5 protein content (*n* = 11) (**b**) measured in whole-muscle lysates in the fed and the fasted state. Data are presented as mean ± SEM

**Fig. 4:**
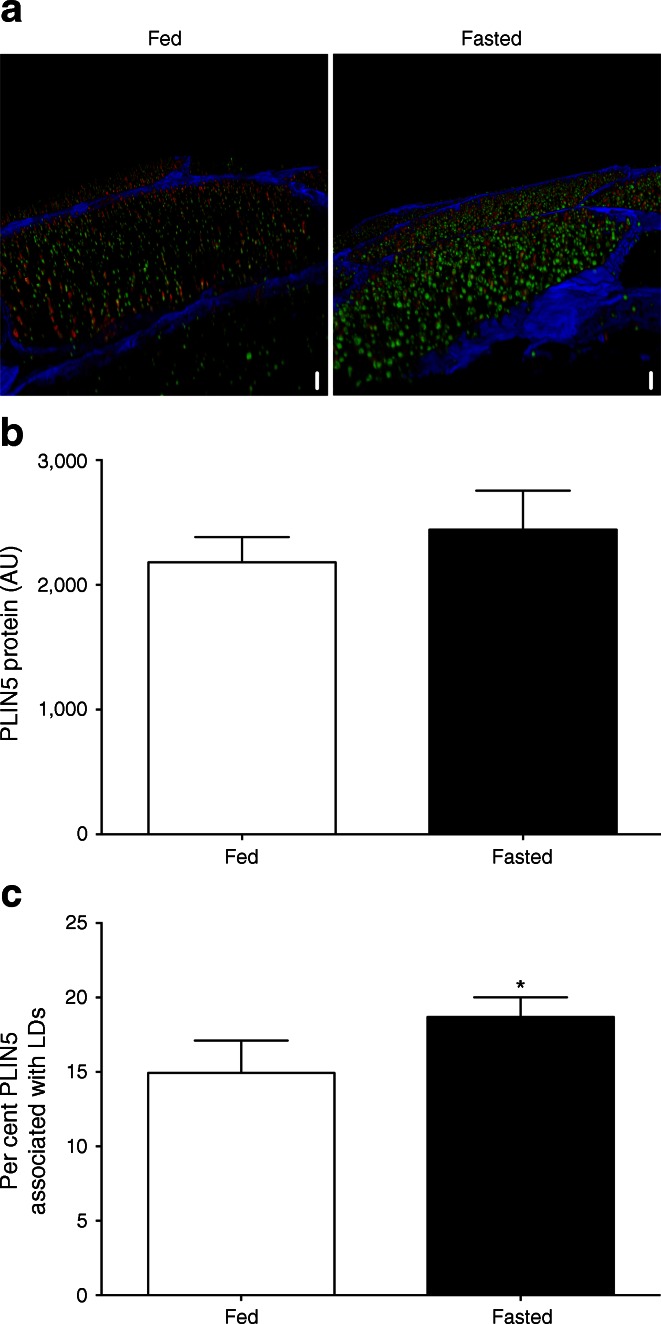
(**a**) Representative three-dimensional images of LD (green) and PLIN5 protein localisation (red) in the fed and fasted state (scale bar, 7 μm). Please visit ESM Videos 1 and 2 for animated three-dimensional reconstruction. (**b**) Average PLIN5 protein content in muscle fibres measured as intensity of PLIN5 staining. (**c**) Percentage of PLIN5 protein content associated with LDs. Data are based upon examination of 3,595 ± 522 and 4,766 ± 489 LDs per participant (*n* = 9) in the fed and fasted state and presented as mean ± SEM, *p < 0.05 vs fed state

Conventional analysis of whole-muscle lysates revealed that fasting had no effect on *PLIN5* mRNA (1.04 ± 0.13 vs 1.11 ± 0.11; *p* = 0.72; Fig. 3a) or PLIN5 protein content (10.4 ± 3.1 vs 10.3 ± 2.8 AU; *p* = 0.99) (Fig. 3b). Muscle lysates were made from sections with equal distribution between type I and type II muscle fibres in the fed and fasted state (50 ± 2% and 52 ± 4% type I fibres in the fed and fasted state, respectively, and 50 ± 2% and 48 ± 4% type II fibres in the fed and fasted state, respectively). PLIN5 is known to be more abundantly expressed in type I fibres. Hence we selected predominantly type I fibres for quantitative high-resolution confocal immunofluorescence microscopy. We simultaneously quantified individual LD size and PLIN5 protein content (Fig. 4a; see ESM Videos 1 and 2 for three-dimensional animated images) by microscopy. In line with the observation in whole-muscle lysates, we observed that total PLIN5 protein content was unaffected by fasting (2,181 ± 202 vs 2,443 ± 312 AU; *p* = 0.276 in the fed and fasted state, respectively, Fig. 4b). To avoid bias introduced by a potential fasting-induced fibre type shift, a similar percentage of type I muscle fibres in the fed (82 ± 6%) and in the fasted state (87 ± 6%) was examined.

Given the putative role of PLIN5 in controlling lipolysis and sequestering fatty acids in LDs by covering the LD surface [35], we examined the fraction of PLIN5 protein associated with LDs. Although total PLIN5 content was not affected by fasting, the fraction of PLIN5 protein directly associated with LDs significantly increased upon fasting from 14.9 ± 2.2% to 18.7 ± 1.3% (Fig. 4c), suggesting a fasting-induced redistribution of PLIN5 from cytosolic sites to the LD surface. Using this novel microscopical approach we could also delineate LDs with high levels of PLIN5 protein (PLIN5+) from LDs devoid of PLIN5 (PLIN5−).

**Fig. 5:**
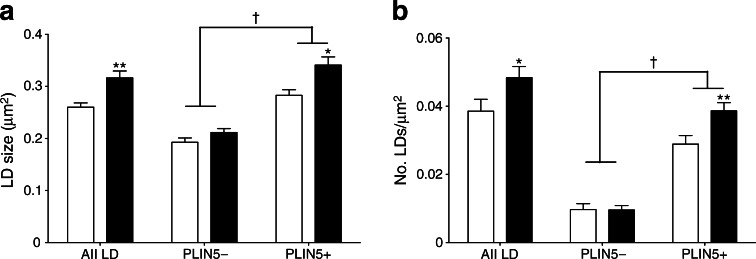
Changes in LD size (**a**) and number (**b**) are accounted for by PLIN5+ LDs. Data are based upon examination of 3,595 ± 522 and 4,766 ± 489 LDs per participant (*n* = 9) in the fed and fasted state and are presented as mean ± SEM, *p < 0.05 or **p < 0.01 vs fed state; ^†^ *p* < 0.001 PLIN5+ vs PLIN5− LDs. White bars, fed state; black bars, fasted state

Upon making the distinction between PLIN5+ and PLIN5− LDs, we observed that the fasting-mediated increase in LD size as well as number was entirely accounted for by PLIN5+ LDs whereas PLIN5− LDs did not change in size or number (Fig. 5a, b). Although these data do not permit statements on causality, they indicate involvement of PLIN5 in the fasting-mediated increase in LD size and number.

**Fig. 6:**
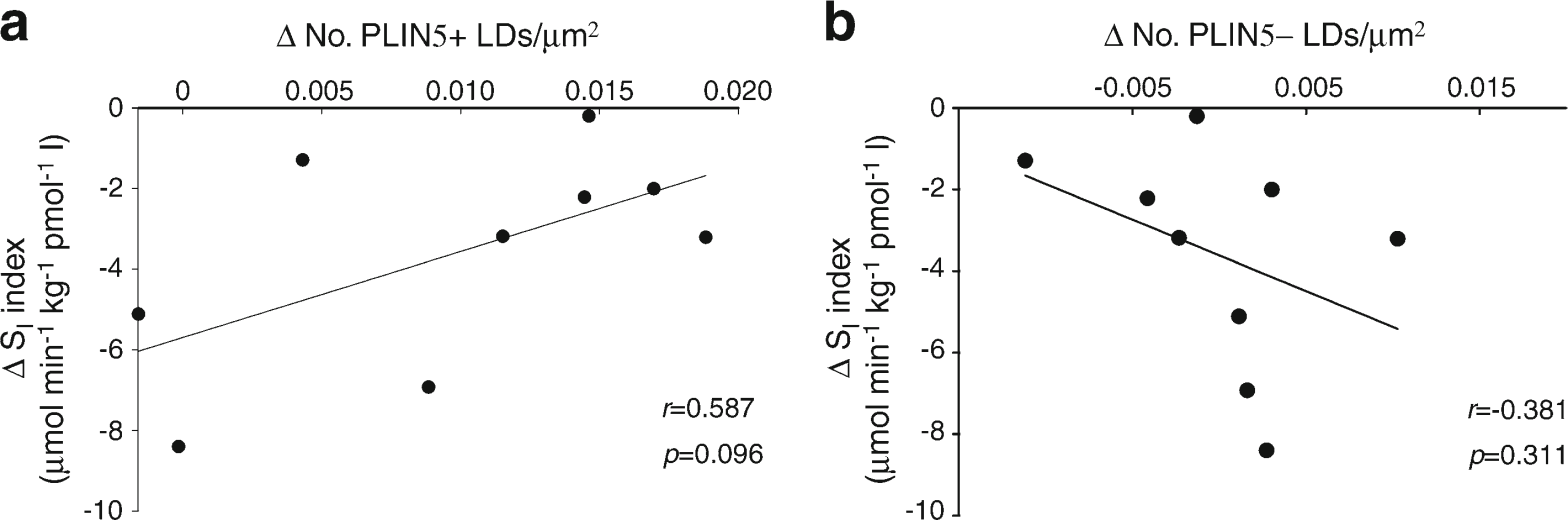
Correlations of the fasting-induced changes (fasted–fed) in the number of PLIN5+ LDs (per μm^2^) (**a**) and the number of PLIN5− LDs (per μm^2^) (**b**) with the reduction in S_I_-index (μmol min^−1^ kg^−1^ pmol^−1^ l) (*n* = 9)

We also observed that the correlation between the change in myocellular fat deposition (change in IMCL) and insulin sensitivity, which was observed upon inclusion of all LDs (*r* = 0.657, *p* = 0.028; Fig. 1), was less strong when only PLIN5+ LDs were taken into account (*r* = 0.587) but still approached the level of significance (*p* = 0.096; Fig. 6a). This was in contrast to LDs devoid of PLIN5, for which this correlation completely vanished (*r* = −0.381, *p* = 0.311; Fig. 6b).

**Fig. 7:**
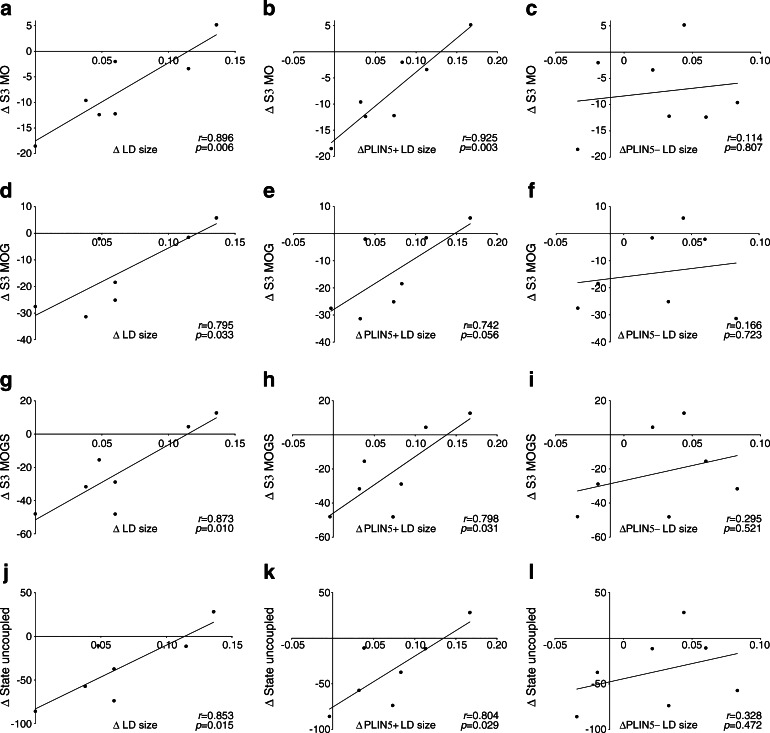
Correlations of the fasting-induced changes in LD size (μm^2^) of all LDs (**a**, **d**, **g**, **j**), PLIN5+ LDs (**b**, **e**, **h**, **k**) and PLIN5− LDs (**c**, **f**, **i**, **l**) with changes in mitochondrial ADP-driven state 3 (S3) respiration (pmol mg^−1^ s^−1^ [mtDNA copy no. × 10^4^]^−1^) in permeabilised muscle fibres on a lipid-derived substrate octanoyl-CoA (MO) (**a**–**c**), octanoyl-CoA + glutamate (MOG) (**d**–**f**) and octanoyl-CoA + glutamate + succinate (MOGS) (**g**–**i**) and during maximal uncoupling (**j**–**l**) (*n* = 7)

Myocellular fat accumulation is not only associated with compromised insulin sensitivity but also with the development of mitochondrial dysfunction [29], a process referred to as mitochondrial lipotoxicity [36]. Using a variety of substrates, we observed that the reduction in mitochondrial function (ADP-driven state 3 respiration, as well as FCCP-mediated maximal uncoupled respiration) upon fasting correlated with the increase in LD size upon fasting (*r* = 0.896, *p* = 0.006 for octanoyl-CoA; *r* = 0.795, *p* = 0.033 for octanoyl-CoA with glutamate; *r* = 0.873, *p* = 0.010 for octanoyl-CoA with glutamate and succinate and *r* = 0.853, *p* = 0.015 for maximal uncoupled respiration; Fig. 7a, d, g and j, respectively). These correlations were maintained if only PLIN5+ LDs were taken into account (Fig. 7b, e, h, k) but, strikingly, were absent for PLIN5− LDs (Fig. 7c, f, i, l).

## Discussion

Here we studied the hypothesis that the LD coat protein PLIN5 increases upon fasting and that PLIN5 is involved in the maintenance of insulin sensitivity under conditions of lipid overload. To this end, we took advantage of a physiological model of insulin resistance in humans—prolonged (60 h) fasting. We showed that the previously reported increase in IMCL upon fasting [29] originates from an increase in both number and size of LDs. Although total PLIN5 content was not affected by fasting, a subcellular redistribution of PLIN5 was observed after fasting, resulting in increased coverage of the LDs with PLIN5. Interestingly, the increase in LD size and number was entirely explained by increases in LD size and number of LDs coated with PLIN5. No such change was observed in droplets devoid of PLIN5. Moreover, we showed that the individuals most capable of increasing muscle fat content upon fasting had the lowest reduction in insulin sensitivity and mitochondrial function. Also, this observation was entirely accounted for by LDs coated with PLIN5. Jointly, these data indicate that the fasting-mediated increase of PLIN5 on the LD surface is part of the adaptive response to fasting to alleviate lipid-induced insulin resistance and to maintain mitochondrial function.

Most of our knowledge on the response of PLIN5 to fasting originates from cardiac muscle in rodent studies [18–20]. These papers indicate that fasting promotes PLIN5 gene expression and protein content in cardiac muscle, whereas in mouse skeletal muscle (gastrocnemius and soleus) PLIN5 protein content appeared to be unaffected by a 24 h fast [18]. The effect of fasting on PLIN5 content in human skeletal muscle has not yet been examined. In line with animal data in skeletal muscle we observed that total PLIN5 content in muscle was unaffected upon fasting. Rather, fasting resulted in redistribution of PLIN5 from cytosolic pools to the LD surface. The fraction of the LDs coated with PLIN5 increased significantly upon fasting, along with an increase in LD size and the fraction of PLIN5 protein content associated with the LD.

Although PLIN5 is best known as an LD coat protein, subcellular fractionation studies revealed the presence of PLIN5 in cytosolic fractions [19]. At present, it is not known whether the cytosolic PLIN5 pool represents unbound PLIN5 or PLIN5 that is somehow located to (yet to be identified) subcellular structures. Hence, it is not known whether the observed redistribution of PLIN5 is in fact a targeted translocation from a defined locus to the LD or whether it represents expansion of nano-scaled LDs coated with PLIN5 that did not exceed the lower detection limit of the microscope in the fed state (and hence were considered cytosolic) but that became detectable upon fasting. It should be noted, however, that fasting resulted in a pool of large-sized LDs decorated with PLIN5 (PLIN5+ LDs) whereas the smaller LDs were devoid of PLIN5. This reduces the likelihood that the pool of cytosolic PLIN5 in fact represents a pool bound to nano-scaled LDs and favours the concept of fasting-mediated redistribution of PLIN5.

In the present model of physiological insulin resistance, PLIN5+ LDs were more numerous and larger than PLIN5− LDs. Hence, LD expansion may originate from coating of the LD with PLIN5 and its inhibitory effect on myocellular LD lipolysis, either by binding to the major triglyceride lipase ATGL [37] or by binding to the co-activator of ATGL, CGI-58 [38]. In trained athletes, high levels of IMCL and high insulin sensitivity accompany high levels of PLIN5 [16, 17], indicating that PLIN5 may modulate insulin sensitivity under conditions of high lipid load. Importantly, delineation of PLIN5+ and PLIN5− LDs for the first time provides experimental support to the notion that increased myocellular fat content does not necessarily impede insulin sensitivity, provided that the excess fat is stored in PLIN5+ LDs. Thus, expanding the pool of LDs decorated with PLIN5 could be considered an adaptive response to fasting to maintain LD dynamics and prevent insulin resistance. Interestingly, we recently showed that individual LDs coated with PLIN5 sequester more bioactive insulin-desensitising lipids than LDs devoid of PLIN5 [39], providing a possible explanation as to why storage of excess fat in PLIN5+ LDs ameliorates lipid-induced insulin resistance.

PLIN5 has also been hypothesised to modulate lipotoxicity by promoting interaction and/or intimate physical association of LDs with mitochondria [21, 40] and to promote oxidative gene expression [40] to facilitate mitochondrial degradation of the fatty acids released from LD lipolysis. Accordingly, PLIN5 may be involved in the prevention of mitochondrial lipotoxicity. In that respect it is of interest to note that the fasting-mediated decline in mitochondrial function (measured as state 3 ADP-driven mitochondrial oxygen uptake and FFCP-mediated maximal uncoupled respiration on a variety of substrates) correlated positively with the fasting-mediated increase in LD size. Upon making the distinction between PLIN5+ and PLIN5− LDs, this correlation appeared to originate from PLIN5+ LDs and was completely absent for PLIN5− LDs for state 3 respiration as well as for maximally uncoupled respiration, irrespective of the substrate used. This again supports the notion that decoration of LDs with PLIN5 may prevent mitochondrial lipotoxicity upon fasting, possibly via expansion of the capacity for inert lipid storage. This notion substantiates our previously reported observation that in muscle of Zucker diabetic fatty rats PLIN5 protein content correlates positively with mitochondrial function [40]. Jointly, our observation that expansion of PLIN5+ LDs ameliorates mitochondrial lipotoxicity and lipid-induced insulin resistance is in line with rodent data indicating a protective role for PLIN5 in lipid-induced insulin resistance [22, 23] and hepatic lipotoxicity [41].

Next to the methodological advance permitting analysis at the level of individual LDs, a major strength of the present study is that, in contrast to most other models of insulin resistance or type 2 diabetes, its design permits conclusions to be drawn on the role of PLIN5 at the very early stages of insulin resistance development. We studied drug-naive, healthy, lean and young men in whom glucose and insulin were well within the normal physiological range. Thus, our observations cannot be attributed to consequences secondary to the pathological state that parallels most models of insulin resistance and hence likely reflect physiological adaptive responses to the very early insulin-resistant state. Maintenance of these adaptive responses may blunt the pathogenesis of insulin resistance.

Myocellular lipid handling is affected by muscle fibre type distribution as well as by sex [42]. In the present study, we carefully considered muscle fibre type distribution as a potential confounder. For data on whole-muscle cell lysates (PLIN5 protein content and gene expression) we ensured equal distribution of type I and type II muscle fibres in the fed and the fasted state. Under untreated and physiological conditions, type I fibres store more lipid and express PLIN5 more abundantly. Hence, we purposely focused our microscopy on type I muscle fibres, with a similar fraction of type I fibres being examined in the fed and fasted state. With respect to sex, it is important to note that in the present study we only examined men. Therefore, extrapolation of the present findings to women must be done with care, especially given the sex differences previously reported for myocellular lipid handling [42], lipid-induced insulin resistance [43], response to fasting [44] and PLIN5 content [45].

In conclusion, the present study shows that PLIN5 protein content did not increase in human skeletal muscle upon fasting, although fasting resulted in a redistribution of PLIN5 from a non-LD-associated cytosolic pool towards the LDs. The ability to store excess fat in skeletal muscle in PLIN5+ LDs upon a prolonged fast associates with blunting of fasting-induced insulin resistance and mitochondrial dysfunction. These data support a role for PLIN5 in the mitigation of fasting-mediated lipotoxicity.

## Electronic supplementary material

Below is the link to the electronic supplementary material.

ESM Video 1 (AVI 39,5516 kb)

ESM Video 2 (AVI 39,5516 kb)

ESM Table 1 (PDF 8 kb)

## Abbreviations

ATGL: Adipose triglyceride lipase
CGI-58: Comparative Gene Identification 58
DGAT1: Diacylglycerol-*O*-acyltransferase 1
FCCP: Carbonyl cyanide-4-(trifluoromethoxy)phenylhydrazone
IMCL: Intramyocellular lipid
LD: Lipid droplet
PLIN2: Perilipin 2
PLIN3: Perilipin 3
PLIN5: Perilipin 5
Rd: Rate of disappearance
S_I_-index: Insulin sensitivity index
