# Supplementary material for: Decoration of intramyocellular lipid droplets with PLIN5 modulates fasting-induced insulin resistance and lipotoxicity in humans
Source: Diabetologia. 2016 Feb 10;59:1040–8. doi: 10.1007/s00125-016-3865-z (PMC4826431; doi:10.1007/s00125-016-3865-z)
Supplement: Supplementary file 3 — (PDF 8 kb) [file 125_2016_3865_MOESM3_ESM.pdf]

Supplemental Table 1: Primer sequences

Gene	Forward	Reverse	Probe
PLIN5 (OXPAT)	GAGCCATGCTGTGGATGTTGTA	CAGTGCCGCGAGCTCTTC	TGGATCACTTCCTGCCCATGACGG
RPLP0	CCATTCTATCATCAACGGGTACAA	AGCAAGTGGGAAGGTGTAATCC	
